# Information Distribution in Multi-Robot Systems: Utility-Based Evaluation Model †

**DOI:** 10.3390/s20030710

**Published:** 2020-01-28

**Authors:** Michał Barciś, Agata Barciś, Hermann Hellwagner

**Affiliations:** Karl Popper Kolleg on Networked Autonomous Aerial Vehicles (KPK NAV), University of Klagenfurt, 9020 Klagenfurt, Austria

**Keywords:** autonomous systems, multi-robot systems, information distribution, utility theory

## Abstract

This work addresses the problem of information distribution in multi-robot systems, with an emphasis on multi-UAV (unmanned aerial vehicle) applications. We present an analytical model that helps evaluate and compare different information distribution schemes in a robotic mission. It serves as a unified framework to represent the usefulness (*utility*) of each message exchanged by the robots. It can be used either on its own in order to assess the information distribution efficacy or as a building block of solutions aimed at optimizing information distribution. Moreover, we present multiple examples of instantiating the model for specific missions. They illustrate various approaches to defining the utility of different information types. Finally, we introduce a proof of concept showing the applicability of the model in a robotic system by implementing it in Robot Operating System 2 (ROS 2) and performing a simple simulated mission using a network emulator. We believe the introduced model can serve as a basis for further research on generic solutions for assessing or optimizing information distribution.

## 1. Introduction

The idea of the presented research is motivated by the communication requirements of a fleet of small autonomous unmanned aerial vehicles (UAVs) realizing a 3D reconstruction of an unknown, global positioning system (GPS)-denied environment without a centralized coordination. UAVs (also denoted as agents, in the following) should explore the given area, locate objects of interest, create 3D models of these objects, and send them to the operator.

Such a mission creates unique communication challenges. It introduces multiple message types, for instance: sensor data (e.g., position, attitude, battery level, etc.), synchronization, mission commands, gathered images, and 3D models. Each of these message types has different characteristics, e.g., varying sending frequency, priority, size, and payload. These characteristics are often heavily dependent on the mission progress and status. For example, exchanging map fragments can be very useful at the beginning of the mission, when all agents explore the environment and contribute new information; conversely, at the end of the mission exchanging map fragments may be of little use, since all agents already know the map and are focused on other mission objectives. Alternatively, two UAVs flying far from each other are not interested in the precise position of each other, but for UAVs operating in close proximity this information might be crucial.

The communication conditions that such a UAV fleet operates in are dynamic: they depend on the proximity of the UAVs, their attitudes, obstacles between them, radio wave propagation, etc. Thus, it is almost impossible to plan the communication a priori and a communication system that adapts to the observed situation is beneficial.

As a first building block of such a communication solution, we present an analytical tool for evaluating information distribution. It allows comparing different communication schemes (or schedules), taking into account the amount of useful information distributed in the system. This model can serve as a tool to compare different systems that realize (and, potentially, optimize) communication in UAV fleets or to provide optimization criteria for such systems.

### 1.1. Concept

The presented solution is directly motivated by the described mission of a multi-UAV system. Dynamic conditions require continuous monitoring of the network status and evaluation of information distribution. Additionally, agile UAVs in a fleet present an interesting challenge in terms of variable goodput: sometimes the UAVs are able to exchange large amounts of data effectively, e.g., sending high-resolution images without problems, but at other times they might even struggle to exchange small messages, e.g., containing their positions. Therefore, it is not enough to handle just the message types that consume the most of the communication fabric; when the communication quality is bad, even the smallest message brings value. This fact supports the need of a unified system that handles messages of all types comprehensively. The presented model addresses these needs. It is worth noting that the motivation and all examples provided in this article are related to UAVs, but that the model may also be useful for other types of multi-robot systems.

Based on the following observations, we introduce an evaluation model for information distribution:

1. A message generated by a sender is meant to provide some information to the receiver, which benefits from this transmission. Hence, our model evaluates the communication from the receiver’s perspective.
2. The usefulness of a message changes (increases or decreases) over time. For example, a map patch received at the beginning of a mission may be useful until the end, depending on whether and when the receiving UAV flies over the map region (useful) or moves elsewhere (not useful). Therefore, a message could add value throughout the whole mission and not only at the time of reception. This usefulness is, however, not known at the time of reception, it depends on the mission progress.
3. The information types can differ significantly and therefore are useful in a different way. For example, knowing the mission objective allows the UAV to plan its next task, whereas getting a new map fragment can help with path planning or localization. Thus, to express the characteristics of each information type it needs to be considered separately.

**Figure 1:**
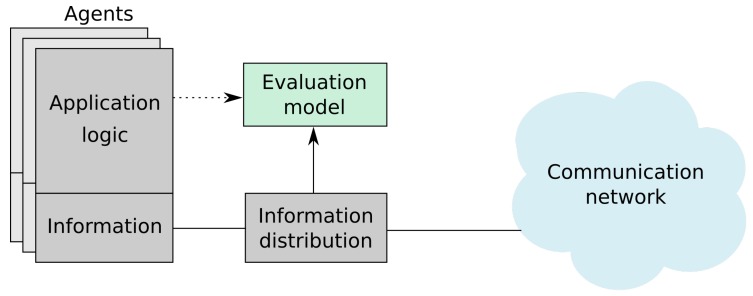
The proposed model aims at abstracting the mission from communication in order to evaluate information distribution.

The model serves as an abstraction between the mission logic and the communication mechanism. This concept is visualized in Figure 1. The model is presented as a block working on top of the information distribution, that separates communication from the information that the agents act upon and exchange. Although the construction of a specific instance of the model is influenced by the application logic, the evaluation does not need to interact with the application during execution, and the only requirement from the communication side is a message-based protocol.

Adapting and using the model to optimize information distribution at flight time is outside the scope of this article.

### 1.2. Novelty

This approach enables a novel way of thinking about information distribution: the utilities provided by the evaluation model could serve as an input to an external algorithm for deciding which messages are more useful (e.g., when optimizing information distribution) or what communication solution performs better (e.g., for comparison purposes). Such an algorithm does not require any additional knowledge about the specific mission or information types in use. It only works on a stream of messages with defined message utility functions. Benefiting from this abstraction, such an external algorithm can be independent of the application and the mission, but at the same time the utilities reflect the value of each message for the mission.

The model only requires knowledge about the received information, which makes it easily applicable in real robotic systems. To that end, the model defines and uses *utility* as a function over time throughout the whole mission, which makes it easier to reason about the usefulness of a given information. For instance, let us analyze a message containing a map fragment that was received by a UAV. It is straightforward to define a function specifying if the UAV was making use (generating utility) of that map fragment at a given point in time, knowing the state of the UAV.

The main contributions of this work comprise the definition of a utility-based model for the evaluation of information distribution schemes (Section 3), descriptions of multiple mission-related communication scenarios together with instances of the evaluation model for these scenarios (presenting the key properties of the model) (Section 4), and a proof of concept showing that the model can be implemented and applied on top of a robotic framework (Section 6).

This work is an extended and refined version of our previous publication [1] presented at the Workshop on Mission-Oriented Wireless Sensor, UAV and Robot Networking 2019 (MiSARN), organized in conjunction with the IEEE International Conference on Computer Communications 2019 (INFOCOM). During the workshop, our work was presented as a first step towards the optimization of information distribution. We have realized that our approach is useful also for other aspects than just optimization of information distribution and can also be used as a separate component. Therefore, we have worked on this extension that is focused on emphasizing and improving the applicability of the model for other works.

The extensions (in the order of importance) include: an extended description of the model (Section 3); addition of a theoretical analysis section (Section 5); a proof of concept demonstrating that the model can be implemented and used on top of a robotic framework (Section 6); a complete overhaul of the related work section (Section 2); and the introduction of an image streaming mission example together with an extension of the model to incorporate data adaptation (Section 4.5).

## 2. Related Work

In this section, we mention the related work from multiple fields: information distribution in robotic systems, Quality of Service (QoS), utility-based approaches and information theory. We observe that most of the related work can be characterized either as widely applicable and generic (e.g., Section 2.2) or focused on a specific application (e.g., Section 2.1). Our goal is to create a model sharing some properties from both of these worlds. It should be generic enough to be used in a uniform way with a variety of information types. At the same time, it should be possible to specialize the model’s behavior for some information types taking into account their most important characteristics for a mission at hand. Because of these unique properties of the introduced model, we did not perform any direct quantitative comparisons with other approaches. Instead, in this section we argue how our approach differs from the state of the art and in Section 5 we present what can be expressed by the model.

### 2.1. Information Distribution

There are many different approaches to information distribution in robotic systems. For instance, Gadd and Newman [2] present a pull-based, centralized solution for map distribution in a fleet of mobile vehicles. They compare multiple methods of choosing the data samples to download based on time and location. Cieslewski et al. [3] address the problem of decentralized data distribution for the simultaneous localization and mapping problem and tackle it by reducing the amount of exchanged data. Mahdoui et al. [4] present a decentralized solution to an exploration problem and reduce communication load by operating on meta information instead of the primary data. Such solutions are very specific and focus on one particular type of information for a particular mission; it is not clear how to generalize such an approach to other information types or how it would perform in other applications.

More generic approaches are also considered. Recently published work by Marcotte et al. [5] presents an algorithm for optimizing communication under bandwidth constraints. The approach assigns a value to each message by simulating the decisions of the agent given that a message was received and without this message. They present an efficient algorithm to compute these values, however it introduces multiple assumptions about the structure of the mission at hand. Best et al. [6] focus on optimizing the data distribution in a multi-robot mission and define the utility of a message as its impact on a mission objective, which is obtainable in the case they are analyzing, but might be hard to estimate in general.

The main difference between the proposed model and the literature mentioned above is that the proposed model just evaluates the data distribution and does not provide any methods to realize it. The most obvious application of the proposed model in this case is to use it in order to compare different solutions and find one that performs the best in the scenario at hand. But the model also addresses both of the problems mentioned earlier in this section: it is generic enough to be used comprehensively with all information types that might be required by an application and it does not need to explicitly know the influence of each message on the mission objective.

### 2.2. Quality of Service (QoS)

Evaluation methods are mostly focused on the typical QoS aspects, such as throughput/goodput, jitter, latency, etc. For instance, Krinkin et al. [7] compare latency and throughput achieved by multiple *Data Distribution Service* (DDS) implementations by sending 100 messages in a row and measuring the reception time of the first and the last message. Similar criteria together with jitter are used by Tardioli et al. [8] in order to compare multiple data distribution algorithms in Robot Operating System (ROS). Martin et al. [9] focus on the influence of security features on latency. Maruyama et al. [10] take into account not only latency, but also examine achieved throughput and introduce multiple capabilities that a communication framework might have (e.g., the ability to send messages of 2 MB in size without data loss using their experimental setup). In a study focused more on real-time applications, Oh et al. [11] use deadline meet ratio as one of the criteria. Tornell et al. [12] perform analytical evaluation of their algorithm and validate the results using experiments. In addition to the delay criterion, they also examine the number of exchanged messages.

In contrast to our proposal, all of the approaches mentioned so far in this section focus on the technical aspects and try to objectively measure parameters of the network. However, exchanging lots of data with minimum latency does not automatically imply that these exchanges are useful. Our approach addresses this problem and aims at capturing the actual value that communication provides for the application at hand.

Many techniques are being developed for measuring Quality of Experience (QoE) (e.g., PEVQ [13] for video streaming). Such techniques are robust and widely used for specific use cases, but it is not possible to generalize them for broader use. They could be incorporated into the proposed model, but on their own they are not able to evaluate the whole information distribution.

By analogy to QoE (and its basis on QoS), we could say that our proposal aims at quantifying the “quality of information” for robots; i.e., the model, while building upon QoS, on a higher level captures the usefulness of information by considering the requirements of robotic interactions and missions.

### 2.3. Utility-Based Approaches

Utility-based approaches have proved to be beneficial in many related fields, including communication. D’Aronco and Frossard [14] present an interesting way to manage network congestions in order to maximize the utility gained by network nodes, with unspecified utility functions. Kimura et al. [15] use utilities in order to solve a message routing problem. They calculate utilities for each pair of nodes and base it on the encounter frequency. Then, they use the computed value in order to make a decision whether to send a message or not. The field of real-time computing also uses utility-based definitions extensively. Raut et al. [16] consider a scheduling problem in which the job’s value deteriorates over time. Li et al. [17] present a heuristic to a similar problem, but using arbitrary utility functions. Gan et al. [18] apply an exponentially decaying utility function in order to address the emergency vehicles scheduling problem.

There are multiple publications on utility-based evaluation of communication, but they focus mainly on multimedia transmissions over the Internet [19,20] and are not directly applicable to robots.

The widespread use of utility functions motivated us to utilize it in our definition of the model. The main difference between our approach and the presented ones is that we calculate the utility continuously at each point in time and integrate it in order to get a total value of utility. This approach is more intuitive to work with and allows us to easily express information value achieved by combining multiple messages.

### 2.4. Information Theory

The field of information theory [21] also considers very similar problems to the ones tackled in this work, but focuses on the amount of information provided by exchanged data and not on the usefulness of this information for the mission at hand. Nevertheless, it was successfully employed in order to estimate the value of communication in a work presented by Williamson et al. [22,23]. The authors also presented the application of their approach using multiple benchmark problems.

In order to use such approaches the coordination algorithms need to be adjusted, which means that it is not possible to apply this approach to all types of information in existing systems. The aim of the proposed model is to create a generic solution to handle all information types. Similarly to approaches presented in Section 2.2, solutions from this section could be incorporated into our model in the definitions of specific information types.

## 3. Model

This section describes the main contribution of this work, i.e., the evaluation model. Most importantly, we present a generic model that must be specified for a mission at hand. We call such a mission-specific definition of the model a *model instance*. In this section, we describe a common framework that all model instances should adhere to. Section 4 presents a variety of simple examples of how to define such instances.

The model’s goal, as presented in this paper, is to compare different communication schemes for a given course of a mission (also called “mission history” in the following) a posteriori. Since the communication is evaluated after the mission is finished, the model treats the mission history as fixed and assigns scores to sets of messages that could have been exchanged. Such an approach implies that we treat the mission history as being independent of the information being communicated and therefore the model is not able to express long-term consequences of not sending or delaying a message. Despite this simplification, given a sound definition of the model instance, the approach is capable of assessing message usefulness (e.g., identifying the most and least important messages) limited to the extent analyzed in Section 5, at the same time abstracting communication from the logic of the system.

### 3.1. Setup

An agent is a process taking part in the information distribution. For instance, an agent could be a communication process of: a UAV, an autonomous rover, a base station operated by humans, or a display visualizing a mission progress.

We evaluate the information distribution during a mission post factum, thus for each agent g∈G we introduce a *history* of a mission $H_{g}$ as a sequence of states of agent *g* during the mission. $H_{g}$ is a function assigning a state of agent *g* to each time *t* during the mission execution; formally, $H_{g}:\left[0,t_{\mathrm{end}}\right]\to S$, where *S* is the set of all possible states and the mission starts at time t=0 and ends at $t=t_{\mathrm{end}}$. A state is a tuple of properties of an agent. We do not define explicitly the structure of the state, as it may vary for each agent and for each mission, but intuitively it represents the knowledge of an agent. Some examples of properties potentially present in the state include: battery level, position, observed map area, etc. In order to successfully exchange a message, the communication protocol might also utilize additional meta data, headers, etc., but the introduced model is focused only on the part of the message that is useful for the application logic.

In order to compare communication schemes, we calculate a numerical value (called *total utility*) U of a set of exchanged messages for a given mission history. Then we compare these values; the higher the utility, the more useful is the received information.

Let M be the set of all messages received by agents and P be the set of all information types. Each message m∈M has at least three properties: m.type∈P is the information type; $m.t_{\mathrm{gen}}\in \left[0,t_{\mathrm{end}}\right]$ is the time when the message was generated; and $m.t_{\mathrm{rcv}}\in \left[0,t_{\mathrm{end}}\right]$ is the reception time. Each message carries some data that can bring different information types. We use the term *generated time* instead of *sent time* in order to emphasize the fact that the actual time when the communication interface has sent the message is not important for the analysis. As a consequence, any delays in sending a message (e.g., because of interference or an ongoing transmission) should influence the final result of the evaluation.

### 3.2. Overview

The utility of the whole system is the sum of all agents’ utilities. Furthermore, the utility of each agent is the sum of utilities of messages containing information of all types received by that agent.

At the core of the model lies the *message utility function*. For each message, it describes how the utility of that message changes as the mission progresses over time. For example, we analyze the utility of the message containing the information about a map fragment for an autonomous UAV. Whenever the UAV flies above the area corresponding to this map fragment, the information is useful and the utility rises. However, as soon as it goes outside of that area, the message is not useful and thus brings no utility at that point in time. When the UAV comes back to this area, the utility of this message rises again. This example presents the continuous nature of the utility functions and their time-dependency. This function is heavily dependent on the mission. Even the same information type could have a different message utility function for different missions.

The utilities of messages containing the same information type are often related to each other. For instance, a UAV could have received a map fragment of the same area twice, in two separate messages. Then, when it flies above this area, not both of these messages are equally useful and they should not bring the same utility: the information is redundant and hence one of the messages should not increase the total utility. A way to aggregate utilities of information of a given type is needed. Therefore, for each information type we define a utility *aggregation function*, which quantifies how useful the transmission of information of a given type was throughout the whole mission. For each time *t*, it takes the utilities of all messages containing the given information type and outputs the aggregated utility for time *t*. The total utility of the given information type is the integral of utility aggregation function over time.

### 3.3. Definitions

The total utility of the system is given by

(1) $$U\left(M\right)=\sum_{g\in G}\hat{U}\left(M_{g},H_{g}\right),$$

where $M_{g}$ is the set of all messages received by agent *g*. $\hat{U}$ is the utility function for a single agent and is defined as

(2) $$\hat{U}\left(M,h\right)=\sum_{p\in P}\omega_{p}\cdot \int_{0}^{t_{\mathrm{end}}}A_{p}\left(M,t,h\left(t\right)\right)\;dt.$$

where $\omega_{p}$ is an importance factor of information type *p*. The arguments *M* and *h* represent the set of messages, and the history of an agent respectively. Note that in Equation (1) this function is used with the arguments $M=M_{g}$ and $h=H_{g}$.

Before defining the aggregation functions we introduce the following notations: $M_{\mathrm{rcv}}^{p}\left(t\right)$ is a subset of set *M*. It includes all messages containing information type *p* received up to time *t*:

(3) $$M_{\mathrm{rcv}}^{p}\left(t\right)=\left\{m\in M:m.t_{\mathrm{rcv}}\leq t\wedge m.\mathrm{type}=p\right\}.$$

It can be thought of as an operator over the set *M* that filters it based on the reception time and information type. Similarly, $M_{\mathrm{gen}}^{p}\left(t\right)$ is a subset of set *M*, including all the messages containing information type *p* generated up to time *t*.

All aggregation functions accept three arguments: *M*, *t* and s=h(t). *M* is the set of messages to be considered, *t* is the time when aggregation takes place and *s* is the state of an agent at time *t*. In this work we introduce three types of aggregation functions: *sum*, *max*, and *last*.

The *sum* aggregation function treats all received messages independently by summing their utilities:

(4) $$A_{p}^{\mathrm{sum}}\left(M,t,s\right)=\sum_{m\in M_{\mathrm{rcv}}^{p}\left(t\right)}U_{p}\left(m,t,s\right).$$

The *max* aggregation function at time *t* takes only the utility of a message with a maximum value:

(5) $$A_{p}^{\max}\left(M,t,s\right)=\max_{m\in M_{\mathrm{rcv}}^{p}\left(t\right)}\;U_{p}\left(m,t,s\right).$$

The *last* aggregation function at time *t* evaluates only the utility for the most recently generated message:

(6) $$\begin{array}{c} m_{\mathrm{last}}\left(t\right)=\underset{m\in M_{\mathrm{gen}}^{p}\left(t\right)}{\arg\;\max}\;m.t_{\mathrm{gen}}, \end{array}$$

(7) $$\begin{array}{c} A_{p}^{\mathrm{last}}\left(M,t,s\right)=U_{p}\left(m_{\mathrm{last}}\left(t\right),t,s\right). \end{array}$$

The *last* aggregation function takes into account the last *generated* message. This allows us to describe situations, when generating a new message invalidates the information contained in a previous message, i.e., as soon as a new message is generated, the previous one does not bring any utility anymore. Unfortunately, it introduces a new challenge for the evaluation of the model from the receiver’s perspective. If a message *m* is generated but not received, we cannot influence the aggregated utility based on the time it was generated. Thus, this kind of utility should only be used in conjunction with a reliable communication protocol.

The defined aggregation functions can be applied to any information type. For example, if we want to use the *sum* aggregation function for information type *test*, we will write: $A_{\mathrm{test}}=A_{\mathrm{test}}^{\mathrm{sum}}$.

## 4. Mission Examples

In this section, we specify the utility model for five different information types in order to provide examples of how the utilities of various information types can differ and how different aggregation functions can be used. The examples are motivated by various research efforts at our university on UAV applications [24] and networking [25]. The choice of examples is not incidental: each of the examples presents a significantly different approach to utility definition. These examples should provide intuition on how the model can be instantiated (i.e., defined for a specific mission) and could serve as a basis to define similar utilities for a variety of information types. We focus our analysis on simplified definitions in order to make them easier to understand and analyze. For certain use cases, where such simplifications are not applicable, more complex definitions could be introduced, e.g., taking into account additional properties of exchanged messages, correctness, or age of information (information decay).

**Figure 2:**
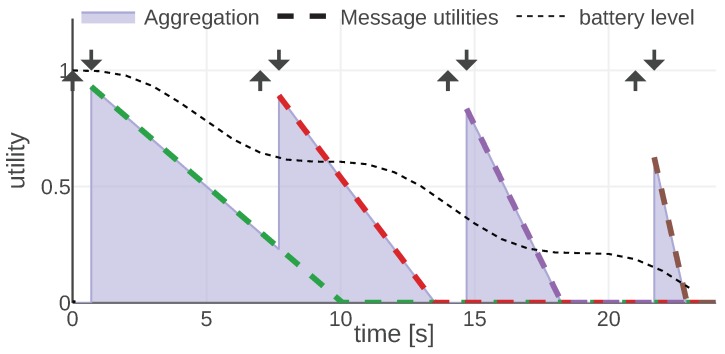
$A_{\mathrm{batt}}$ and $U_{\mathrm{batt}}$ functions plotted for an instance of the given mission. © 2019 IEEE. Reprinted, with permission, from [1].

For each information type, we present an example of the mission scenario and a plot of the message utility and aggregation functions. As shown in Figure 2, all of them follow the same convention: message utilities are plotted with dashed lines, each message is represented with a different color. The aggregation function is plotted with a solid line and the area under its plot is filled. Additionally, each moment when a message was generated is marked with an arrow pointing up and the reception time is marked with an arrow pointing down.

### 4.1. Agents’ Properties

In this section we analyze two types of information, both of them representing robots’ properties: battery level and position in 2D. The definitions of utility of messages containing information of these types, depend on the way the information provided by these messages is used. For example, consider using the position of another robot to estimate how far it is from a given point. We can assume this distance in the worst case will change linearly over time, based on the maximum speed of the robot. However, if we use the last received position in order to estimate where exactly the agent is now, the probability of estimating the exact position, based only on the maximum speed and the last received position decreases quadratically over time in the 2D case (the agent can go into any direction on a plane) and cubicly over time in the 3D case (it potentially travels in any direction in space).

This distinction in the interpretation of position data serves as a good illustration of one of the most important properties of the introduced model: the ability to abstract communication from mission logic. Once the utility functions are defined, the evaluation model allows us to treat all the messages in exactly the same way, independently of the way the message is used by the mission.

#### 4.1.1. Battery Level

We assume agents are exchanging messages containing their battery levels. The algorithm running on each agent directly uses the data from the most recent message and assumes it to be correct, even if it is outdated. From this message, it extracts the information about the sender’s availability, i.e., if the sender’s battery is depleted, it is no longer available.

In order to introduce the message utility function for this scenario, we define ρ as a pessimistic battery depletion rate, i.e., a rate that is higher than the maximum possible battery consumption rate. Hence, the battery level after time $\hat{t}$ drops in the worst case by

(8) $$R_{\mathrm{batt}}\left(\hat{t}\right)=\hat{t}\cdot \rho .$$

The utility of a message reflects the ratio between a pessimistic real battery level at a given time and the message data.

Here, the *max* aggregation function is used, because the algorithm running on an agent always uses the most useful message, which in this case is always the most recent message.

The message utility and aggregation functions for the battery information are defined as follows:

(9) $$\begin{array}{c} U_{\mathrm{batt}}\left(m,t,s\right)=\max\left(0,1-\frac{R_{\mathrm{batt}}\left(t-m.t_{\mathrm{gen}}\right)}{m.\mathrm{batt}\_\mathrm{level}}\right) \end{array}$$

(10) $$\begin{array}{c} A_{\mathrm{batt}}=A_{\mathrm{batt}}^{\max} \end{array}$$

By studying Figure 2 presenting the plot of Equations (9) and (10) for an example instance of the given mission, we can observe some properties of these utilities. First of all, a message is useful for the receiver, starting from its reception time to the time when the battery would be completely depleted in the worst case (assuming pessimistic depletion rate). The utility decreases over time as soon as the message is generated. This is based on the intuition that we have the perfect information only at the time of measurement. The more time has passed, the less precise information we have about when the battery is going to deplete. Additionally, we can observe that the utility falls faster when the measured battery level is low.

#### 4.1.2. Position

In this example, the agents exchange position information. This position information is handled in a similar way as the battery level information, with two small differences. First, instead of using a battery depletion rate, we define the area where the agent can be after time $\hat{t}$ as

(11) $$R_{\mathrm{pos}}\left(\hat{t}\right)=\pi v_{\max}^{2}\cdot {\hat{t}}^{2}.$$

Second, we define an area *A*, which could either be the maximum area of a mission or an area big enough that knowing the agent is somewhere in that area does not bring any valuable information. The utility of a message over time is defined as a ratio between the complement of the area where the agent can be and *A*. Such a definition maintains the following property: if the exact position of an agent is known, the utility equals 1 and it drops to 0 when there is no information about the agent’s position (it can be anywhere in *A*). Similarly to the battery level example, the position information also uses the *max* aggregation function:

(12) $$\begin{array}{c} U_{\mathrm{pos}}\left(m,t,s\right)=\max\left(0,1-\frac{R_{\mathrm{pos}}\left(t-m.t_{\mathrm{gen}}\right)}{A}\right) \end{array}$$

(13) $$\begin{array}{c} A_{\mathrm{pos}}=A_{\mathrm{pos}}^{\max} \end{array}$$

**Figure 3:**
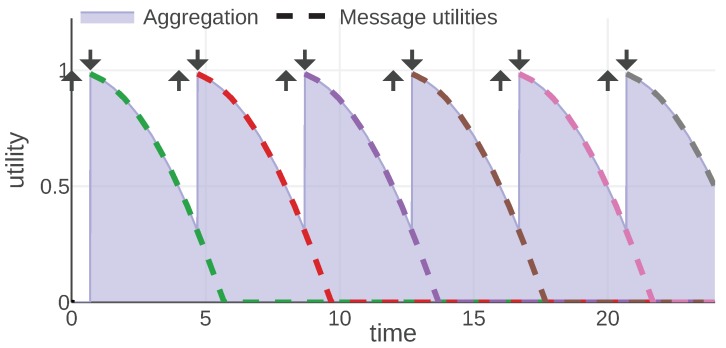
$A_{\mathrm{pos}}$ and $U_{\mathrm{pos}}$ functions plotted for a mission example. © 2019 IEEE. Reprinted, with permission, from [1].

Equations (12) and (13) are plotted in Figure 3. The result is very similar to the battery type, but the utility decreases quadratically over time. Additionally, the utility is independent of both the message content (last known position) and the state, so the shape of the message utility function is always the same.

### 4.2. Mission Status Commands

During a mission, it might be required to communicate a status of the mission to all agents involved in it. Examples of statuses include: *waiting for mission start*, *mission in progress*, *abandon mission*, *mission finished*. The main characteristic of this type of information is that, as soon as the new message is generated, the mission status changes and all the old messages are not valid anymore. We express that with a constant message utility function and the *last* aggregation function:

(14) $$\begin{array}{c} U_{\mathrm{status}}\left(m,t,s\right)=\left\{\begin{array}{cc} 1 & \mathrm{if}\;t>m.t_{\mathrm{rcv}}\\ 0 & \mathrm{otherwise} \end{array}\right., \end{array}$$

(15) $$\begin{array}{c} A_{\mathrm{status}}=A_{\mathrm{status}}^{\mathrm{last}}. \end{array}$$

**Figure 4:**
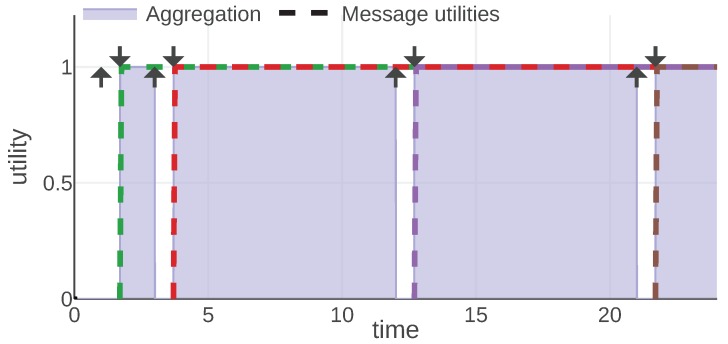
$A_{\mathrm{status}}$ and $U_{\mathrm{status}}$ functions plotted for a mission example. © 2019 IEEE. Reprinted, with permission, from [1].

Figure 4 presents a plot of the aggregation and utility functions for an example communication scenario. An agent aggregates the utility when it knows the current mission status. Each delayed message means that the agent does not know the current status; thus, the final utility is decreased. In the figure this shows as a lack of aggregated utility between the generation and reception of each message. Note that the utility of each message never drops, e.g., the utility of the green message is equal to 1 for almost the whole mission. It is the aggregation function that decides which message should be considered at which point in time and introduces the areas of no utility.

It can be easily observed that, for this information type, a communication scheme that minimizes transmission delays is preferred. The fact that the communication is evaluated from the receiver’s perspective makes such a definition possible—the sender would not have access to information about delays without any additional protocol.

Because the *last* aggregation function is being used, the approach is only useful to evaluate the performance at the end of the mission assuming a reliable communication channel. Sometimes it might be beneficial to compute the utilities live, while the mission is in progress. Unfortunately, because of the receiver-centric nature of the model, in that case we cannot use the generation time of a new message, because that message might not have been delivered yet. Instead, we can change our modeling approach and define utility as a probability that the status of the mission did not change since the message we have received was generated.

We assume that changes of the status of a mission could be modeled as a Poisson point process, i.e., a process where events (in this case status changes) appear independently at a constant average rate λ. Then, a time between events is modeled using exponential distribution. From a memorylessness property of that distribution we know that we can also use it to express a distribution of a time between an observation of a status (i.e., the time when a message is generated) and a change of that status. So the probability that an event occurred since the last observation is expressed using a cumulative distribution function (CDF) of exponential distribution: $1-e^{-\lambda t}$. We are interested in an inverse of this event (i.e., that the status did *not* change), so we can define the utility as:

(16) $$\begin{array}{c} U_{{\mathrm{status}}^{\prime }}\left(m,t,s\right)=e^{-\lambda (t-m.t_{\mathrm{gen}})}, \end{array}$$

(17) $$\begin{array}{c} A_{{\mathrm{status}}^{\prime }}=A_{{\mathrm{status}}^{\prime }}^{\max}. \end{array}$$

**Figure 5:**
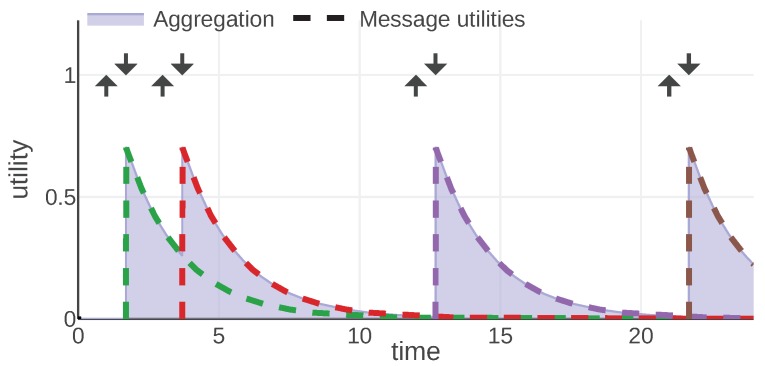
$A_{{\mathrm{status}}^{\prime }}$ and $U_{{\mathrm{status}}^{\prime }}$ functions plotted for a mission example. © 2019 IEEE. Reprinted, with permission, from [1].

We use the *max* aggregation function, because we are interested in the message that gives us the maximum probability that a status did not change since it was received. These functions are plotted in Figure 5 for an example of a mission with λ=0.5. We can observe that the utility falls down rapidly. Therefore, an information of this kind should be repeated periodically to increase the probability that it is still valid.

### 4.3. Mission Objectives

Mission objectives, although conceptually similar to mission status, are distinctively different from them. Examples of objectives include: *explore sector A*, *monitor sector B*, *map sector C*. They define goals to be pursued during the mission by agents.

We say that an agent is *working on* an objective if it actively tries to fulfill it. For instance, if it received a message *explore sector A*, immediately it does not receive any utility, but it knows that traveling through this sector is beneficial for the mission. We say that it starts to work on this objective as soon as it decides to go to this sector and explore it and this is the time when the utility of this message rises. However, it does not necessarily mean that the agent has to explore the whole sector; it can contribute some work and then continue with a different objective.

This example demonstrates that the message utility is related to the agent’s state (in this case with respect to the objectives that the agent is trying to fulfill) and that it varies over time. It is worth noting that the utility of the message is not necessarily decreasing over time; in this case, it will be equal to 0 at the time of reception and will rise as soon as the agent decides to work on the objective.

An agent cannot start the work on an objective before a message introducing this objective is received by it. It is, however, possible that an agent will work on multiple objectives at the same time. Therefore, we will treat the utilities associated with each objective independently. Hence, the *sum* aggregation function is used.

For the sake of simple presentation, we assume all objectives have equal importance and each unit of time spent working on the objective is equally useful. Thus, we define the utility of a message that contains objective *o* as 1 whenever the agent is actively working on objective *o* and 0 otherwise:

(18) $$\begin{array}{c} U_{\mathrm{objective}}\left(m,t,s\right)=\left\{\begin{array}{cc} 1 & \mathrm{if}\;m.o\in s.O_{\mathrm{pursued}}\\ 0 & \mathrm{otherwise} \end{array}\right., \end{array}$$

(19) $$\begin{array}{c} A_{\mathrm{objective}}=A_{{\mathrm{objective}}^{\prime }}^{\mathrm{sum}} \end{array}$$

where m.o is the objective introduced in message *m* and $s.O_{\mathrm{pursued}}$ is a set of objectives on which the agent was actively working in state *s*.

The assumptions about equal importance and constant utility are not required. For some missions it might be more practical to assume the message utility is given by a decreasing function (e.g., if joining at the beginning makes an agent more useful). We could also assign higher utilities to the more important objectives.

**Figure 6:**
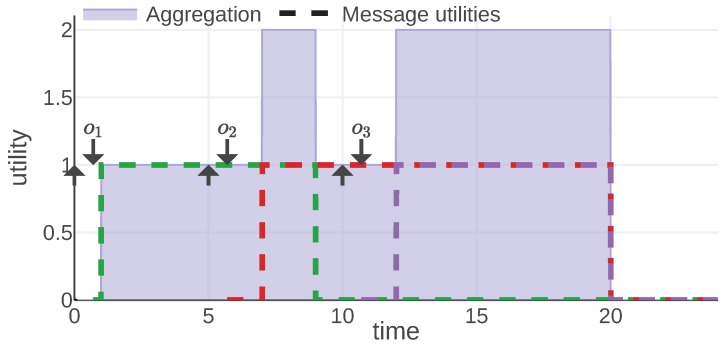
$A_{\mathrm{objective}}$ and $U_{\mathrm{objective}}$ functions plotted for a mission example. © 2019 IEEE. Reprinted, with permission, from [1].

Figure 6 presents a plot of aggregation and message utility functions for an example communication scenario. In this scenario, the agent was working on three objectives: $o_{1}$ between time 1 and 9, $o_{2}$ between time 7 and 20 and $o_{3}$ between time 12 and 20. During time periods [7,9] and [12,20] the agent is working on two objectives simultaneously. The aggregated utility at each given time is the number of objectives the agent is working on at that time.

If the message corresponding to a particular objective is not received, the agent does not know about it and cannot work on this objective. The utility will not be generated. Therefore, a communication scheme that prioritizes the objectives that are more important to be worked on by the agent will perform well in the evaluation framework.

### 4.4. Localization Using a Map

The next type of information analyzed in this work is a map used for localization. We assume each message *m* contains a sector of a map covering area $m.A_{\mathrm{map}}$. Each agent maintains a union of all of the received sectors and stores the area of this union in its state as $s.A_{\mathrm{map}}$. We will call this union a *gathered map* of an agent. At each point in time an agent observes the area $s.A_{\mathrm{obs}}$ around it and tries to localize itself on the gathered map. The bigger the intersection between the observed area and the gathered map, the better the localization result.

We define the added area of message *m* as the difference between $m.A_{\mathrm{map}}$ and all the areas previously received:

(20) $$N_{m}=m.A_{\mathrm{map}}\backslash \left(\underset{\begin{array}{c} m^{\prime }\in M_{\mathrm{rcv}}^{\mathrm{loc}}\left(m.t_{\mathrm{rcv}}\right)\\ m^{\prime }\neq m \end{array}}{⋃}m^{\prime }.A_{\mathrm{map}}\right).$$

The utility of a message *m* at time *t* equals the ratio of the common part of the observed area and $N_{m}$ to the whole observed area. The proposed model is presented in Equations (21) and (22).

(21) $$\begin{array}{c} U_{\mathrm{loc}}\left(m,t,s\right)=\frac{N_{m}\;\cap \;s.A_{\mathrm{obs}}}{s.A_{\mathrm{obs}}}, \end{array}$$

(22) $$\begin{array}{c} A_{\mathrm{loc}}=A_{\mathrm{loc}}^{\mathrm{sum}}. \end{array}$$

**Figure 7:**
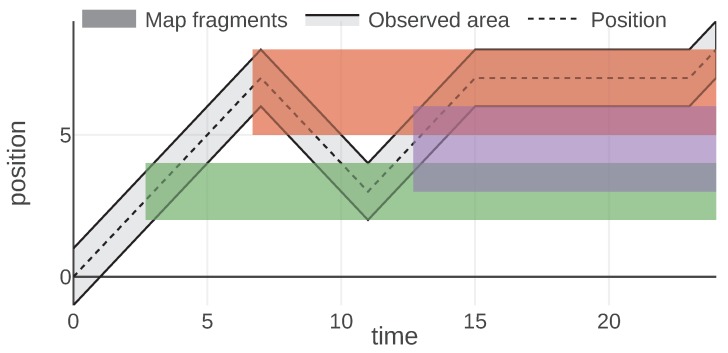
Plot of the agent position and the received map data in 1D. © 2019 IEEE. Reprinted, with permission, from [1].

**Figure 8:**
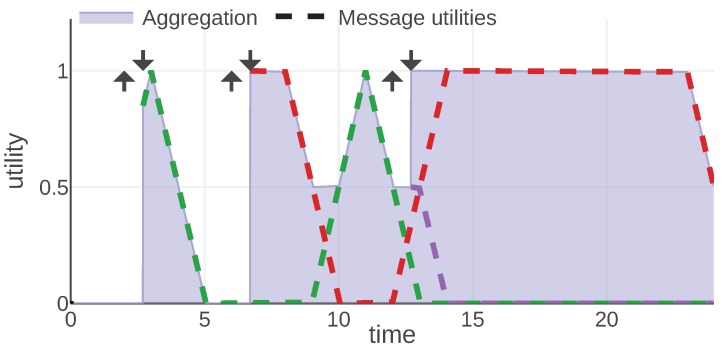
$A_{\mathrm{loc}}$ and $U_{\mathrm{loc}}$ functions plotted for a mission example. © 2019 IEEE. Reprinted, with permission, from [1].

An example of a mission introduced to analyze this information type is visualized in Figure 7. The plot visualizes the 1D position of an agent over time with a dashed line. Its observed area is marked with a light gray color and the borders of this area are plotted with a solid black line. During a mission, the agent received three map fragments, at times 2.7, 6.7 and 12.7. These messages are visualized with a filled rectangle of different colors representing the area of a 1D map associated with the message. The fragment is valid indefinitely, hence the area is marked since the reception time until the end of the mission. The color of each map fragment matches the color of a message utility function plotted in Figure 8.

In Figure 7 and Figure 8 we can observe how message utilities can change throughout a mission. For instance, the first map fragment (drawn in green) is useful just after the reception, then at time 5 it brings no utility since the agent cannot observe this sector anymore. Around time 9 the agent comes back and the map fragment is useful again. These figures also present nicely the properties of the model, namely that the utility of information is state-related (in this case it depends on the agent’s position and thus its observed area) and that utility of each message is independent of the utilities provided by other messages. The third map fragment (drawn in purple) overlaps with previously known areas, thus, bringing utility only for the newly introduced part. This demonstrates how the possible redundancies of the information can be handled.

### 4.5. Image Streaming

Another interesting kind of information that could be distributed between agents is a stream of images, for instance being captured by a camera of one of the agents. The analysis is motivated by a surveillance mission, where multiple agents (e.g., drones) are monitoring an area and taking pictures of some targets. They exchange these pictures in order to offload some computations and share the knowledge about targets. We focus on situations, where a ground personnel may connect to such drones in order to preview the exchanged image stream. We assume each message contains a single image. We call a stream of such messages viewed by humans a video.

Clearly, there is a wealth of techniques aiming at optimizing video streaming performance. The role of this model is to evaluate those approaches. The simplified example presented here focuses only on one aspect of Quality of Experience (in this case a perceived frame rate) but it could be used as a basis for more complete solutions. In this article, the image streaming information type serves as an opportunity to demonstrate a different approach to defining the utility model instance. So far, message utility functions were defined in a structural fashion, i.e., we were arguing about the amount of information that each message brings at a time instance and constructing the functions accordingly. This time, we first specify some properties of the resulting total utility and based on that we come up with a form of message utility function.

We base our definition of the model on an assumption that the human perception of a frame rate of a video can be modeled by the Weber–Fechner law [26]. The law relates the subjective perception of human senses (*p*) to the objective stimulus intensity (*f*) and claims that this relationship is logarithmic:

(23) $$p=\alpha \ln\frac{f}{f_{0}}$$

for some α and $f_{0}$. $f_{0}$ is a smallest stimulus that can be perceived.

In our case, the observed subjective quality is the smoothness of the video and an objective measure is expressed in frames per second (FPS). The utilities provided by the model should be proportional to the perceived quality, hence in the definition of the model we aim at having the logarithmic relationship between utilities and FPS. Therefore the model should have the following property:

(24) $$U\left(W_{f}\right)=\hat{\alpha }\ln\frac{f}{f_{0}}$$

where $W_{f}$ is a message set which results in a video being streamed at *f* FPS, formally:

(25) $$W_{f}=\left\{\;m|m.t_{\mathrm{gen}}=\frac{n}{f}\wedge n\in N\;\right\}.$$

Even though in the original formulation of Fechner’s law (Equation (23)) the natural logarithm is used, we can change the base of the logarithm by substituting $\hat{\alpha }=\frac{S}{\log B}$, where *B* is a new base of the logarithm and *S* is the parameter of the model. We have decided to use B=2, which is often used in IT applications. The final form of the utility function is:

(26) $$U\left(W_{f}\right)=S\log_{2}\frac{f}{f_{0}}$$

Next, we introduce a message utility function and we show that the obtained model fulfills the requirement defined in Equation (26). For the sake of simple presentation, in this analysis we assume the absence of delays (i.e., the frame is received at the same time it is being generated). We take delays into account in the definition of message utility, so they will lead to a lower total utility value, but we do not examine their impact. Naturally, we use the *max* aggregation function since the last frame always brings the most useful information. Let us assume a message utility function has the following form:

(27) $$U_{\mathrm{vid}}\_\mathrm{ideal}\left(m,t,s\right)=-\log_{2}\left(t-m.t_{\mathrm{gen}}\right)k+l$$

By solving a system of Equations (26) and (27) we can find out that the proposed model indeed results in a pursued relationship for k=S and arbitrary *l*. Unfortunately, this definition is impractical: it might result in an infinitely large or negative utility value, which might mean that receiving some messages introduces loss or disproportionately huge gains.

Therefore, we have decided to limit the values of Equation (27) by capping them to [0, 1], while still using the *max* aggregation function. It results in the following model:

(28) $$\begin{array}{c} U_{\mathrm{vid}}\left(m,t,s\right)=\max\left(0,\min\left(1,-\log_{2}\left(t-m.t_{\mathrm{gen}}\right)k+l\right)\right), \end{array}$$

(29) $$\begin{array}{c} A_{\mathrm{vid}}=A_{\mathrm{vid}}^{\max}. \end{array}$$

The maximum value of total utility per second is limited to 1, by such an approach. We assign this value to an exchange that results in a video streamed with frame rate of $f_{\max}$ FPS. Any exchange that results in a higher frame rate should receive the same utility, formally:

(30) $$\forall f:f>f_{\max}.\;U\left(W_{f}\right)=1.$$

If we had tried to pursue the same approach as we did with the $U_{\mathrm{vid}}\_\mathrm{ideal}$ (Equation (27)) utility function, i.e., tried to solve a system of Equations (26), (28) and (30), there would have been no solutions. The model received in that case no longer results in the logarithmic relationship we were pursuing. However, if instead of looking for solutions for any *f* in Equation (26) we pick just one frequency $f_{1}$, we get the following results:

(31) $$\begin{array}{c} l=1+\log_{2}\left(\frac{1}{f_{\max}}\right)k \end{array}$$

(32) $$\begin{array}{c} k=\frac{S}{1-\frac{f_{1}}{2f_{\max}\ln2}} \end{array}$$

In practice it means that the relationship is not logarithmic anymore. In the “ideal” case, each time the frequency was divided by *B*, the utility was lowered by *S*. In the new model this property holds only for a specific frequency $f_{1}$. However, in practice it should not be a problem, as usually the system will aim at streaming videos close to some predefined frame rate and we will be evaluating exchanges that result in frame rates similar to that one. Additionally, the relationship we have achieved is indeed very close to logarithmic.

**Figure 9:**
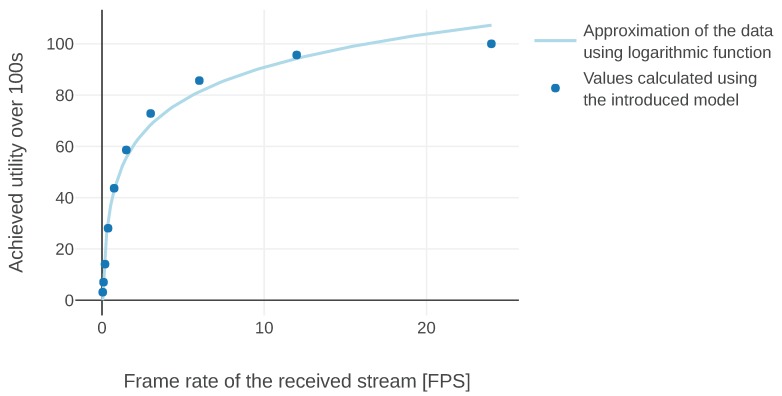
Total utility achieved by evaluating message exchanges which resulted in different constant frame rates using the introduced model.

We have calculated the values of parameters *k* and *l* for S=0.1, $f_{\max}=24\;\mathrm{Hz}$, and $f_{1}=12\;\mathrm{Hz}$. Then, we have simulated missions in which images were sent with different frame rates over 100 s. In practice, it means that we were evaluating the model for different $W_{f}$. The utilities generated during these simulations are presented in Figure 9. The blue line represents an ideal model while circles correspond to utilities achieved by simulating the modified version. We find the resulting model satisfactory, because it is motivated by an abstraction of human perception, which itself often does not strictly adhere to the logarithmic relationship.

**Figure 10:**
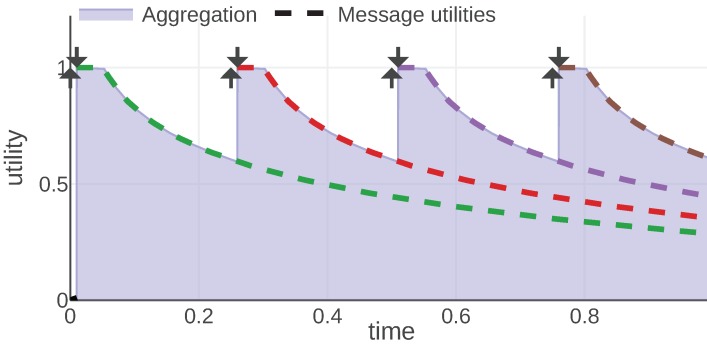
$A_{\mathrm{vid}}$ and $U_{\mathrm{vid}}$ functions plotted for a video streaming mission example.

Figure 10 presents a plot of the utility and aggregation functions from the introduced model. We can observe that exactly for $\frac{1}{f_{1}}$ the function is flat. If messages were to be exchanged with a higher frequency than $f_{1}$, the result will just be a constant function. After that time period, the function decreases logarithmically. The slope of that decrease can be adjusted with the *S* parameter.

In the following paragraphs, we examine the possibility to include source adaptation in the model. Most types of data can be adapted in order to reduce the amount of data sent while sacrificing the information content. A video stream is a good example, because image compression techniques can achieve a very good compression rate while the stream still looks good for a viewer. Of course, an important part of video adaptation is reducing its frame rate. However, this part is already taken into account by the model, as it assigns lower utilities when the message (an image) is not received, therefore penalizing lower FPS streams. In the following, we will only examine how to incorporate into the model the information about a lower quality of a transferred message (e.g., a lower resolution).

We model adaptation in the following way. First of all, we define the notion of a *version of a message*. Each message could be available in multiple versions. They are all generated at the same time and they all represent the same knowledge, but the information content might differ, i.e., for any message, a sender can decide to send an adapted (e.g., lower quality) version of that message. We call “the *best version* of a message” a version that has the highest information content. For instance, in the case of a video stream it could mean that the same image can be sent in multiple resolutions. The best version of that message will be the image with highest resolution available for the sender.

The evaluation model should take the existence of those versions into account and assign lower utilities to versions of messages that contain less information. At any point in time the sender can decide to re-transmit the same message in a different version (e.g., in a higher resolution). As soon as a better version is received, the evaluation model should use it instead of the previous one.

In order to implement these features, we make two changes to the model introduced in Section 3. First of all, the model did not include the notion of versions of messages. Hence, in the case of the *max* aggregation function, we redefine each message in the model as being the best version of that message at a given time (all other versions are not used). It means that the definition of $M_{\mathrm{rcv}}^{p}\left(t\right)$ from Equation (3) changes and should contain only the best versions of all messages available at time *t*. Second, we need to introduce a version of the message utility function that takes adaptation into account. Our example is based on video streaming messages, because it uses the image resolution, but a similar parameter of a message can be used for any other type. The adaptation is realized by changing the resolution of images. We assume the utility of the adapted image is proportional to its diagonal, expressed in number of pixels. The message utility function takes the following form:

(33) $$\begin{array}{c} U_{{\mathrm{vid}}^{\prime }}\left(m,t,s\right)=\frac{m.d}{d}\cdot U_{\mathrm{vid}}\left(m,t,s\right), \end{array}$$

where m.d is a diagonal of the image provided in message *m* and *d* is a diagonal of a frame in a maximum available resolution on the sender’s side.

## 5. Theoretical Analysis of the Model

In this section, we demonstrate the universality of the model by addressing the question of what communication history can be assessed and compared by the model. Obviously, it depends on the definition of the instance of the model (message utilities for a given information type), but we focus only on the general formulation of the model (Equations (1) and (2)) and analyze what constraints are introduced by this definition.

We start by showing how to define an instance of the model in order to mimic the behavior of other evaluation models that just assign a single numerical value to each message. Then we analyze a bit more complicated and general case of message filtering. In relation to the mission examples introduced in Section 4, the first subsection presents a generalized use case that is much easier to express than the presented mission examples. On the other hand, the second subsection argues about use cases that are harder (or even impossible) to express than the presented mission examples.

In this section we introduce multiple theorems, proofs of which are provided in Appendix A.

### 5.1. External Message Utility Value

Sometimes the message utility is already given as a numerical value, for instance, by another evaluation algorithm that is specialized for a given information type. Each message then gets a single value assigned to it and the values of all exchanged messages are summed up. This is a very simple scheme that might often be useful, but some situations cannot be captured by it; for example, it cannot capture applications in which the utility of one message depends on the successful transmission of another message. It might still be useful to incorporate such a simple scheme into our model, e.g., in order to have one unified model for all information types or to use an optimization technique that is based on this model. Let us assume there is a function *V* that assigns scores to messages and we would like to define a model instance for information type *p* that preserves the scores of *V*. This could be achieved using the following message utility function:

(34) $$U_{p}\left(m,t,s\right)=V\left(m\right)\cdot \delta \left(t-m.t_{\mathrm{rcv}}\right)$$

where δ is Dirac delta.

Such definition allows us to integrate state-of-art evaluation methods that are specialized for some type and are specifying the utility of a message as a single value. Moreover, this approach could be easily modified to take into account the state of the robot at the time of reception, delay of the message, etc. It means that creating an instance of the model for the whole system at hand (i.e., for all information types) might be simplified. For instance, let us assume there is a system that exchanges drones’ positions, coordination information, and video stream between UAVs and our goal is to create an instance of the introduced evaluation model for such a system. Good evaluation algorithms for coordination information and video steam might already be available. We could integrate them into the system using the solution proposed in this section. The only thing left would be to define message utilities for UAVs’ positions and weights for each type.

### 5.2. Message Filtering

Dropping some messages is one of the most efficient ways to reduce the amount of data exchanged in a system. In this section we mark the set of all potential messages as M. An evaluation model should be able to help us identify a subset of a predefined number of messages of M that we can drop with minimal performance loss.

More specifically, given any order $\leq_{P(M)}$ on subsets of M (i.e., filtered messages), one should be able to construct a version of the model that is able to represent it: the resulting total utilities should maintain the same order. We also handle separately one corner case: no set can be lower than an empty set. It is reasonable: rarely we would prefer receiving no information at all throughout the whole mission over any message.

This intuition is formalized using the equation:

(35) $$\forall M.\;\forall \leq_{P(M)}.\;\forall M^{\prime },M^{\prime \prime }\subseteq M.\;\exists U.\;M^{\prime }\leq_{P(M)}M^{\prime \prime }\Rightarrow U\left(M^{\prime }\right)\leq U\left(M^{\prime \prime }\right)$$

Another way to interpret this equation is to introduce an adversary knowing all potential messages M and telling us that some particular subset of exchanged messages is better than another. The adversary specifies a set of such relations between two subsets, which together form the order $\leq_{P(M)}$. Our role is to find such an instance of the utility model U that maintains the order for each pair of subsets given by the adversary.

Unfortunately, the following theorem is true:

**Theorem** **1.** *Equation *(35)* does not hold in general.*

Because of the receiver-centric design of this model, it is not possible to freely order subsets that differ in messages received by multiple agents. For instance, consider a mission in which UAVs are photographing an area and sending images to two base stations. It does not matter to which base station a UAV sent a message, but there is no use of sending a photo of the same area to both base stations. Such situations cannot be expressed using the presented model. In real life, we can get around this problem: the base stations could exchange between themselves some meta-data about the area already photographed and based on this the utility of images could be reduced.

Even though there is a family of orders that cannot be expressed using the model, we formulate the following theorem that specifies what can be expressed:

**Theorem** **2.** *Equation *(35)* holds with the following constraint:*

(36) $$\forall M^{\prime },M^{\prime \prime }\subseteq M.\;\left|\left\{\;m.\mathrm{receiver}|m\in M^{\prime }▵M^{\prime \prime }\;\right\}\right|>1\Rightarrow M^{\prime }{≰}_{P(M)}M^{\prime \prime }$$

*where *△* is the symmetric difference of two sets, i.e., for any sets A and B,* A▵B=(A\B)∪(B\A) *.*

This theorem means that the order $\leq_{P(M)}$ can only compare subsets in which all of the changed were received by the same agent—messages received by other agents need to stay the same. Specifically, the theorem implies that we can express all situations in which an optimal solution for the whole group of agents could be made by choosing an optimal solution for each agent independently. It also shows that the model can describe all systems in which each agent is aware only of his own knowledge and does not know what messages were received by other agents.

The obtained result assures that even with the added constraint, the model can describe a variety of situations and should be adjustable to any mission at hand. Note that the results are achieved with an unspecified aggregation function. As soon as we introduce some specific aggregation to the model, its expressive power can be vastly reduced in exchange for usability and simplicity granted by that aggregation. In summary, the results mean that for each situation there exists a combination of aggregation functions that gives the model high expressive power.

## 6. Model Application

The aim of this section is twofold: (1) it shows the usefulness of the model by employing it in a simple mission, and (2) it presents a proof of concept demonstrating a way to integrate the presented model into a robotic system. Additionally, the section could serve as an example of how similar models (used for optimization or for evaluation) can be tested and show what technologies are available.

In order to present the applicability of the model, we show how it can be used to address a real-life problem related to UAV communication. In our scenario, a UAV performs some mission and it needs to periodically report its battery level to the ground station. It has two ways to achieve this: either by sending it over a direct radio link or by using the cellular mobile network and sending the data “over the Internet”.

The possibility of evaluating the model in a real robotic mission is shown by implementing it on top of a popular robotic framework in a virtualized environment that is as close as possible to a real one. A robotic platform was not used because we wanted to present the performance of the model in extreme communication conditions, which might be very hard to control in the real world.

We assume the following characteristics have the most influence on the communication: the Internet connection is reliable (all messages are delivered), but introduces some variable delay; the direct connection has a constant low delay of 10 ms, but sometimes it does not deliver a message. The UAV can actively measure these characteristics, i.e., it knows the current message delay and drop rate. Based on them, it can choose which communication channel should be used for sending messages. In order to make this decision, it uses the introduced evaluation model.

The model for battery information is specified as in Section 4.1.1 with the pessimistic battery depletion rate equal to 1% of full capacity per second. The simulated mission takes 100 s and during that time the actual battery level depletes much more slowly than in the pessimistic case. We assume a linear depletion starting from 100% and reaching 88% at the end of the mission.

The implementation of our example mission logic is very simple: each agent is periodically publishing its battery status once per second. All of the other agents are subscribed to receive these messages. At the end of the mission, the devices are calculating the accumulated utility and saving it for further analysis.

### 6.1. Experiment Setup

The proposed model is able to evaluate any exchange of information implemented using any message-based communication protocol and provided all agents use a synchronized time. Such low requirements make the model applicable for many technologies, e.g., built on top of a publish–subscribe communication paradigm, using pull-based approaches, etc. The experiment setup was designed in order to emphasize these properties by making use of frameworks and technologies popular in practical robotic applications and it allows us to directly use the same software on real hardware. At the same time, it allows us to control the communication conditions in order to run reliable and repeatable experiments.

We use *Docker* (https://www.docker.com/) as a virtualization technology. It improves the realism of the system because processes running on different agents have very limited interference capabilities. Additionally, it forces the solution to work on separate (virtualized) hosts, which from the implementation perspective is very similar to an actual multi-robot system.

In order to be able to examine a wide spectrum of parameters, the experiments were performed using a network emulator. This allows us to adjust network parameters like communication delays or packet drop rate and run thousands of tests automatically. At the same time, we benefit from being able to use exactly the same communication solutions as in a real robotic system. The use of a network simulator (like *NS-3* (https://www.nsnam.org/)) was also considered, but it is hard to integrate such a simulator with popular robotic frameworks; eventually, the aim of this example is to showcase the applicability of the introduced model for robotic systems.

The network emulator used in the presented experiments is a fork of *Mininet* (http://mininet.org/) with added support for Docker containers: *Containernet* (https://containernet.github.io/). As wireless connections are not supported in Containernet, we decided to model the network in our scenario as a router to which all devices (drones and a base station) are connected (a star topology). In the described case, this simplification is acceptable, because the single communication line between the router and the base station assures only one packet can be transmitted at a time. In real life, channel access methods used in both LTE and 802.11 serve a similar role as this router. The link bandwidth was always set to the minimum available value of 1 Mbps.

The simulation framework developed by us first starts a Docker container for each agent in the system, connects them through a virtualized network, and runs a single instance of the experiment. When it detects the end of the experiment, it shuts down all containers and cleans the network configuration. It is also possible to run multiple experiments in parallel. In order to avoid high CPU usage, which might make the results less reproducible, we limit ourselves to run 8 experiments at a time. After each experiment all outputs from the programs are stored together with a log of all captured packets and the experiment’s metadata. This data can be then further analyzed.

The experiment running on each agent was implemented in *Python* and *Robot Operating System 2 Crystal Clemmys* (https://index.ros.org/doc/ros2/) with two *Data Distribution Service* (DDS) implementations being tested: *eProsima Fast RTPS* (https://github.com/eProsima/Fast-RTPS) and *RTI Connext DDS* (https://www.rti.com/).

The analysis of the results was conducted using Python in *Jupyter Notebook* (https://jupyter.org/) and *Wireshark* (https://www.wireshark.org/). The results are visualized with the *Plotly* (https://plot.ly/) plotting library.

### 6.2. Results

**Figure 11:**
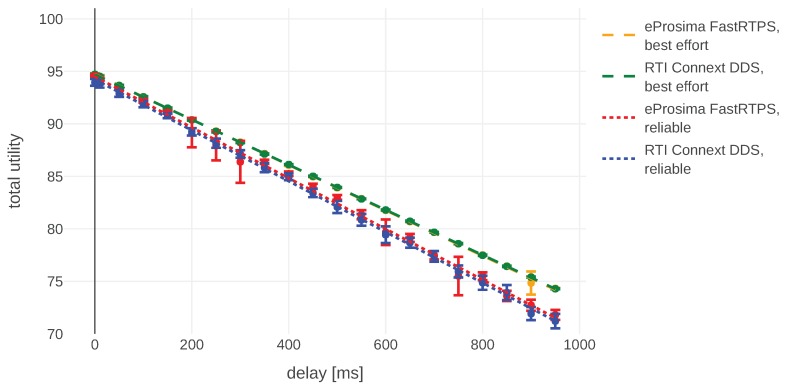
Total utility aggregated during the simulation of the exemplary mission (100 s) for connections with different delays.

**Figure 12:**
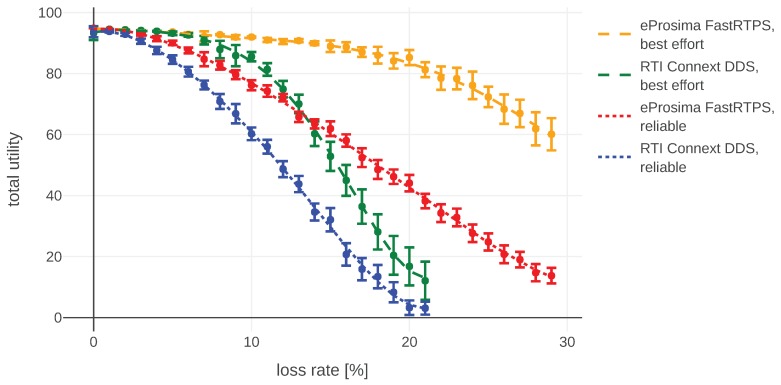
Total utility aggregated during the simulation of the exemplary mission (100 s) for a lossy connection. The experiments with Connext data distribution service (DDS) with loss rate higher than 21% were unfeasible, because the discovery phase was consuming most of the mission time, therefore the communication between robots was often not established at all.

Even though the main goal of this section is to show a simple example of how the model can be applied in real life, we decided to use the opportunity and also see how the changes in our setup influence the final outcome. The simulation-based study was conducted in order to compare performance with different number of agents, DDS configurations, and DDS implementations. All of them are run with the assumption introduced at the beginning of this section: small messages are being exchanged relatively rarely (once per second) in two types of experiments simulating different communication channels:

(1) with drop rate varying between 0 and 0.3 and constant delay, presented in Figure 11; and
(2) with drop rate equal to 0 and delay in the range [0,1] seconds, presented in Figure 12.

In both plots, error bars represent 95% confidence intervals and dots mark mean utility values for a given experiment configuration. For each sample point we run at least 50 experiments, often more. The variance of number of experiments is caused by the fact that during some experiments (especially done using lossy setups) the connection was not established at all. We have not included those results in our analysis, i.e., we analyzed only the situations were initial connection was successful.

At the beginning we elaborate about the implementation- and configuration-specific results without getting into details what the specific setting does. Interested readers can find the explanations in the DDS specifications and manuals of the corresponding implementations. At the end of this section we present more generic results, not bound to our configurations, together with ideas how they could be applied.

We compared the following *Quality of Service* (QoS) settings for both DDS implementations in our mission: reliability, durability, and history. The reliability setting allows us to choose between a reliable and best-effort connection. In the best-effort case, each message will just be sent once and no efforts will be taken to re-transmit it. The reliable setting re-transmits messages and tries to ensure all of them will be delivered in the right order. The durability parameter decides if publishers should remember old messages and send them to late-joining subscribers. The history setting allows us to choose how many past messages are kept in memory for re-transmissions. Changing any of both durability and history did not yield any observable difference. This is probably due to the small packet size compared to available throughput, which means that all messages can be delivered as soon as possible and there is no need to prioritize them. Reliable connection always introduces some overhead. Hence, it has slightly worse performance than best effort in scenarios where dropping some single messages does not influence the mission a lot. These results can be observed in Figure 11 and Figure 12.

A larger number of agents taking part in the mission on the other hand has a huge effect on the time it took for the setup of the connections between agents. Both DDS implementations are using custom discovery protocols to establish connections between participants. Even with as few as 8 agents, it could be observed that this phase is consuming a significant part of the mission time (around a minute), whereas for 3 to 4 agents it was almost instant (a couple of seconds). This result was expected, because the discovery protocol needs to exchange $O(n^{2})$ messages, where *n* is the number of agents, and was in fact observed by us also in a real-world experiments using robots in one of our previous works [27]. However, after the discovery period is finished the system runs flawlessly for any number of agents between 1 and 10, which is explained by the small amount of data exchanged during the mission. No experiments were conducted with more than 10 devices, because a long discovery period (over a minute) for both implementations of DDS made it impractical to run a huge number of experiments. It is possible to decrease the discovery time by tweaking the parameters of DDS implementations, but all the experiments were done using the default values and higher number of devices taking part in the mission does not contribute much to the main goal of described experiments.

We have identified some differences between DDS implementations. In this particular scenario FastRTPS was doing a bit better than Connext, especially over a lossy connection. This is probably caused by smaller packet sizes used by FastRTPS and therefore a lower chance of packet loss. For example, in order to send a message containing a battery level by FastRTPS in a best-effort mode, it transmits 334 bytes “on wire”. Connext DDS for the same message utilizes 774 bytes. In both cases, packets contain mainly protocol-specific information. Those differences can be observed in Figure 11 and Figure 12. We are reluctant to make any claims about the performance of these implementations based on these results, because of the small message sizes and the fact that the differences start to show up only when packet loss is higher than 0.1.

**Figure 13:**
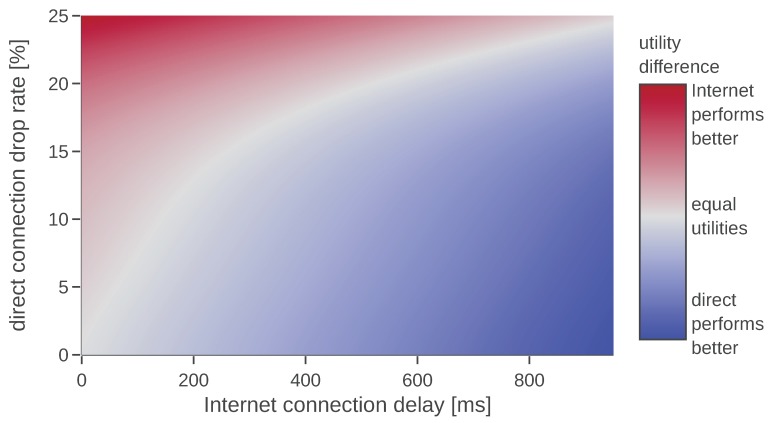
Results of the simulation of an exemplary mission.

Based on the presented results, we can construct a 2D plot that visualizes the difference between the utilities of direct and Internet connection. An example of such a plot is given in Figure 13. In order to create it we have used the parameters that achieved the best performance, i.e., best-effort communication using FastRTPS. With the blue color (lower right corner) we mark configurations in which the direct connection has higher utility than the Internet connection; deeper blue means bigger difference. The red color (upper left corner) represents that the Internet connection has higher utility. The generated data could be used by a UAV to decide which communication channel to use.

## 7. Conclusions

Motivated by the fact that the usefulness of a message changes over time and different information types are useful in a different way, we have introduced a utility-based model for evaluation of information distribution in multi-robot systems. The proposed model provides the following benefits: First, it is able to handle different types of exchanged messages and information conveyed by these messages in a uniform way. This allows us to evaluate information distribution independently of the mission using a unified framework. Second, it provides a layer of abstraction between the mission and algorithms working on the information distribution scheme (e.g., evaluating or optimizing it), providing a common representation of the usefulness of each message for the mission. Third, given the receiver-centric approach, it takes into account only the received messages. This makes the model applicable to real-life robotic systems, even if the communication channel is not reliable. Fourth, it does not depend on the feedback about the message reception, thus it does not cause any additional overhead in the network.

Furthermore, the presented application of the model demonstrates its usefulness and serves as a proof of concept, showing how the evaluation model can be integrated into robotic systems and which technologies can be used in order to achieve that.

We believe this work will serve as a solid foundation for a variety of research topics (from a theoretical analysis of different communication solutions to practical applications in multi-robot systems), including the online use of the model in order to optimize communication.

Other interesting topics for future work might include an effort to categorize utility functions in order to facilitate model definition for system engineers. Stretching the idea even further, future work could consider research aiming at automatic definition of message utility functions based on mission objectives or even based on data gathered during mission execution (in the spirit of reinforcement learning).

Extensions of the model could also be considered. The fact that the information might have been corrupted during communication could be taken into account. An interesting extension could include an assumption that the agents are not cooperating and some of them should not receive some information.

## Abbreviations

The following abbreviations are used in this manuscript:

1D, 2D, 3D	1, 2, 3 Dimensions
CPU	Central Processing Unit
FPS	Frames Per Second
GPS	Global Positioning System
KPK	Karl Popper Kolleg
LTE	Long-Term Evolution
UAV	Unmanned Aerial Vehicle
NAV	Networked Autonomous Aerial Vehicle
QoE	Quality of Experience
QoS	Quality of Service
ROS	Robot Operating System

## Appendix A. Proofs of Theorems from Section 5

**Proof of Theorem** **1.** Let us consider the following four subsets of M:

$$\begin{array}{c} M^{I}=M_{1}\cup M_{2}\\ M^{II}=M_{1}\\ M^{III}=M_{2}\\ M^{IV}=\emptyset \end{array}$$

where $M_{1}$ and $M_{2}$ are sets of all messages received by, subsequently, the first and the second agent. We will show that the ordering

$$M^{I}\geq_{P(M)}M^{II}\geq_{P(M)}M^{IV}\geq_{P(M)}M^{III}$$

cannot be expressed using the model. The model sums up utilities of all agents, so it can be simplified:

$$U\left(M\right)=\sum_{g\in G}X\left(M_{g}\right)$$

for some function *X*. This means that the utility function has to have the following form for the considered sets:

$$\begin{array}{c} U\left(M^{I}\right)=x_{1}+x_{2}\\ U\left(M^{II}\right)=x_{1}+p\\ U\left(M^{III}\right)=x_{2}+p\\ U(M^{IV})=2p, \end{array}$$

for some $x_{1}$, $x_{2}$ and *p* (note that p=X(∅)). By applying Equation (35) we get:

$$x_{1}+x_{2}>x_{1}+p>2p>x_{2}+p,$$

therefore

$$x_{1}+x_{2}>x_{1}+p\Rightarrow x_{2}>p,$$

but

$$2p>x_{2}+p\Rightarrow p>x_{2},$$

which leads to contradiction. □

**Proof of Theorem** **2.** For each agent g∈G there exists a monotone function $f_{g}:P\left(M_{g}\right)\to R$ that fulfills the following requirements:

(1) P(X) is a power set of *X*,
(2) $f_{g}$ should preserve the order $\leq_{P(M)}$ on its domain.

We know such a function exists because $\leq_{P(M)}$ is a partial order on P(M). Observe that all $f_{g}$ have disjointed domains, therefore we can construct a function f:P(M)→R that uses appropriate $f_{g}$ functions for parts of its domain:

(A1) $$f\left(M\right)=\sum_{g\in G}f_{g}\left(M_{g}\right),$$

$M_{g}$ is a subset of *M* containing only messages received by agent *g*. We set all information type weights ω to 1 and use the following aggregation function:

(A2) $$A_{p}^{\mathrm{proof}}\left(M,t,s\right)=\delta \left(t\right)\frac{f(M)}{\left|P\right|}$$

where δ is Dirac delta. We calculate the utility given this aggregation function:

(A3) $$U\left(M\right)=\sum_{g\in G}\sum_{p\in P}\omega_{p}\cdot \int_{0}^{t_{\mathrm{end}}}A_{p}^{\mathrm{proof}}\left(M_{g},t,\emptyset \right)=\sum_{g\in G}f\left(M_{g}\right).$$

Because $M_{g}$ contains only messages received by agent *g* we can simplify it as:

(A4) $$U\left(M\right)=\sum_{g\in G}f_{g}\left(M_{g}\right).$$

Given this definition of the model let us consider any $M^{\prime }$ and $M^{\prime \prime }$ such that $M^{\prime }\leq_{P(M)}M^{\prime \prime }$. Lets define $G_{▵}=\left\{\;m.\mathrm{receiver}|m\in M^{\prime }▵M^{\prime \prime }\;\right\}$, which contains all agents for which there are different messages in sets $M^{\prime }$ and $M^{\prime \prime }$. Based on that definition, we can observe that

(A5) $$U\left(M^{\prime }\right)=\sum_{g\in G\backslash G_{▵}}f_{g}\left(M_{g}^{\prime }\right)+\sum_{g\in G_{▵}}f_{g}\left(M_{g}^{\prime }\right)=\sum_{g\in G\backslash G_{▵}}f_{g}\left(M_{g}^{\prime \prime }\right)+\sum_{g\in G_{▵}}f_{g}\left(M_{g}^{\prime }\right).$$

Because of the constraint introduced in Equation (36), we know that the set $G_{▵}$ has zero or one element. If it has zero elements, then:

(A6) $$U\left(M^{\prime }\right)=\sum_{g\in G\backslash G_{▵}}f_{g}\left(M_{g}^{\prime }\right)=\sum_{g\in G\backslash G_{▵}}f_{g}\left(M_{g}^{\prime \prime }\right)=U\left(M^{\prime \prime }\right)$$

If there is exactly one element in $G_{▵}$, we can mark it as $\hat{g}$. Because $f_{\hat{g}}$ maintains the order $\leq_{P(M)}$ we deduce that

(A7) $$f_{\hat{g}}\left(M_{\hat{g}}^{\prime }\right)\leq f_{\hat{g}}\left(M_{\hat{g}}^{\prime \prime }\right).$$

By applying this result to Equation (A5) we finally see that

(A8) $$U\left(M^{\prime }\right)\leq U\left(M^{\prime \prime }\right).$$

□
